# Racial Differences in Sleep Outcomes in the First Two Years of College

**DOI:** 10.1007/s40615-026-03091-y

**Published:** 2026-07-16

**Authors:** Tiffany Yip, Kyle Lorenzo, G. Alice Woolverton, Jiawei Wu, Heining Cham, David Chae, Mona El-Sheikh

**Affiliations:** 1 Department of Psychology, Fordham University, New York City, NY, USA; 2 Department of Psychology, St. Mary’s College of Maryland, St Marys City, MD, USA; 3 Department of Pediatrics, Brigham and Women’s Hospital, Boston, MA, USA; 4 Harvard Medical School, Boston, MA, USA; 5 Department of Social, Behavioral, and Population Sciences, Tulane University, New Orleans, LA, USA; 6 Department of Human Development and Family Studies, Auburn University, Auburn, AL, USA

**Keywords:** Sleep, Young adults, College transition, College students, Longitudinal

## Abstract

This study investigated trajectories of ethnic and racial sleep disparities during the first two years of college, focusing on several dimensions of sleep. Ethnically and racially diverse first-year college students (*n* = 451; Asian = 19%, Black = 15%, Latinx = 19%, multiracial = 23%, White = 24%; female = 76%, male = 22%, non-binary = 2%) wore wrist actigraphs for two weeks every semester for four semesters. Findings suggest that across different sleep dimensions (duration, bedtime, rise-time, and efficiency), ethnic and racial disparities emerge over time. White students had the most improvements in sleep over time, with increases in sleep duration and no change to their sleep efficiency. During this period, Black and Latinx students woke up later, white and multiracial students went to bed earlier, and Asian students had no change in bedtime or risetime. All racial and ethnic minority students declined in sleep efficiency. These findings document persisting ethnic and racial sleep disparities among diverse college students across a range of sleep dimensions and have implications for targeted interventions to improve sleep outcomes for diverse youth.

## Introduction

### Sleep Health in College Students

Sleep is central to physical health, emotional well-being, and academic performance, making it especially relevant for college students who are navigating increased academic demands and developmental stress [1, 2]. However, many students experience notable sleep difficulties. New academic pressures, irregular schedules, and shifting social routines are common as students adjust to college and are linked to compromised sleep health [3, 4]. Large-scale studies consistently document substantial rates of poor sleep quality among college samples; for example, over 60% of students in a U.S. sample reported poor sleep [5], and similar rates have been reported in other countries, such as Brazil [6]. These prevalence findings are particularly concerning because numerous studies link poor sleep quality to worse downstream physical and psychological health, diminished daily functioning, and worse academic performance [2, 5, 7]. Given that college coincides with a developmental period when many mental health disorders first emerge, health behaviors become more firmly established, and academic demands intensify, persistent sleep problems during this time may have cascading consequences for both immediate functioning and longer-term health. Accordingly, there is a need for research that more precisely characterizes sleep health in college populations.

### The Multidimensional Nature of Sleep Health and Objective Measurement

Sleep is increasingly understood as a multidimensional construct that includes timing, duration, quality, regularity, and daytime functioning [8]. Because these components may diverge, such as when adequate sleep duration coexists with poor sleep efficiency, scholars emphasize the importance of examining multiple dimensions of sleep simultaneously to capture the full complexity of sleep [8, 9] A multidimensional framework also facilitates more nuanced identification of which aspects of sleep are most affected in particular populations. Guided by this perspective, the present study examines sleep across several domains: sleep duration, bedtime-related variables (bedtime, risetime), and sleep efficiency. Assessing multiple domains of sleep provides a more comprehensive picture of sleep health than relying on any single indicator [10] and can yield nuanced insights into the particular sleep-related challenges that college students experience. In turn, this level of specificity can inform the development of targeted supports and interventions that address the dimensions of sleep most disrupted in this population.

Actigraphy is particularly well-suited for capturing this multidimensional profile of sleep health, as it provides objective estimates of sleep timing, duration, regularity, and nighttime activity across extended periods with minimal participant burden [11]. By continuously recording sleep–wake patterns in students’ natural environments, actigraphy enhances the ecological validity of sleep assessment and allows for the detection of subtle differences across multiple sleep domains [12]. Actigraphy has demonstrated stronger validity than self-report measures for estimating sleep timing and duration, making it a valuable tool for improving the accuracy of multidimensional sleep measurement in college students [11, 13].

### Sleep Trajectories Across the College Years

The transition from high school to college represents a major developmental shift that often disrupts previously stable routines and sleep patterns. Changes in living arrangements, reduced parental structure, increased social opportunities, and later bedtimes frequently produce more variable sleep–wake schedules during this transition period [14, 15]. Research consistently shows that first-year college students experience shorter sleep duration, increased sleep irregularity, and more insufficient sleep compared to their pre-college years [5, 14]. These disruptions appear to reflect the combined influence of expanded autonomy, shifting sleep schedules, and heightened academic and social demands characteristic of the move to college [5, 14, 16]. Recent work tracking students across their first semester of college also finds substantial variability and instability in sleep timing, duration, and quality during this transition, suggesting that students’ sleep patterns may be highly unsettled during the initial adjustment to college [17].

Beyond the initial shift into college, sleep continues to change across the undergraduate years, although the direction and magnitude of these changes vary across studies. Some longitudinal work indicates that sleep duration declines and sleep schedules become more irregular as students progress through college. For example, one study followed students for four years and found gradual declines in sleep quantity, later bedtimes, and worsening sleep quality from freshman to senior year, suggesting increasing dysregulation over time [18]. Other multi-semester studies similarly report persistent short sleep duration and fluctuations in sleep quality across academic terms [19, 20]. However, not all research indicates uniform deterioration. Large multi-institution surveys reveal considerable variability, with some students showing stable or even improved sleep quality as they settle into college routines [16]. By contrast, work on insomnia symptoms shows that students who enter college with higher levels of sleep difficulties or psychological distress tend to experience persistent or worsening problems with sleep onset and sleep quality across the undergraduate years [21]. Taken together, the evidence is mixed regarding how sleep health changes during the college years, and notably, no existing longitudinal research has tracked college students’ sleep using actigraphy or examined multidimensional sleep profiles across more than one academic year. As a result, little is known about how objective sleep patterns shift across the early undergraduate years.

### Ethnic and Racial Disparities in Sleep

Further, understanding longitudinal sleep patterns in college students requires considering how sleep health may differ across racial and ethnic groups, given well-documented disparities in sleep health [22–24]. Research with racially and ethnically diverse samples frequently shows that white individuals report more favorable sleep outcomes than their minoritized peers [23, 25]. Evidence from adolescent studies indicates clear differences in sleep duration, with Black and Latinx youth obtaining the least sleep, Asian youth reporting intermediate levels, and white youth obtaining the most [24, 26]. Comparable trends appear in college populations: for instance, Black students have been shown to report shorter sleep and poorer sleep quality relative to white students [27]. Additional work has found that racially and ethnically minoritized students are more likely than white students to experience short sleep during the initial weeks of college [28].

Research with adult populations shows parallel patterns, with racially and ethnically minoritized adults regularly reporting poorer sleep quality than their white counterparts [29–31]. However, other studies find no significant racial differences, suggesting that the presence of disparities may depend on the specific social or developmental context being examined [31]. Because the college environment represents a distinct context with its own academic, social, and structural demands, it is important to determine whether similar variability in racial and ethnic sleep patterns is evident among students over the course of college, particularly as campus diversity continues to increase [32]. Clarifying these patterns is especially important given that sleep plays a central role in students’ academic performance and long-term health, making it critical to understand how racial and ethnic differences in sleep develop across the college years and how they can inform efforts to support diverse student populations [14].

### Current Study

More longitudinal research exploring sleep across multiple years using objective measures of multidimensional sleep health is needed. In addition, little research has examined longitudinal changes in ethnic and racial sleep disparities among racially diverse college students while considering multiple dimensions of sleep. Longitudinal research is essential for determining whether students’ sleep improves, declines, or remains stable as they progress through college, which dimensions of sleep are changing, and whether these patterns differ by racial group. Understanding how sleep unfolds developmentally, and whether racial disparities widen, narrow, or remain stable, can help identify when students are most vulnerable and which groups may require targeted support. Examining multiple sleep dimensions over time also clarifies whether specific aspects of sleep health follow distinct patterns that contribute to emerging disparities, helping to pinpoint the developmental periods and sleep processes most responsive to intervention.

The current study uses wrist-actigraphy to investigate semester-by-semester changes in sleep within the first two years of college and possible ethnic and racial sleep disparities. The first aim is to characterize sleep trajectories across the first two years of college. The second aim is to examine whether these trajectories differ by ethnic and racial group membership. Consistent with prior findings [23], it is hypothesized that sleep health will improve overall during the first two years of college and that sleep disparities will persist over time, with white students showing greater improvements in sleep health compared to ethnic and racial minorities. Although differences in sleep health among ethnic and racial minority groups have been observed [24–26] existing findings are mixed, so the pattern of possible differences across specific sleep dimensions in the current sample is considered exploratory.

### Methodology

#### Sample

This study draws from a 5-year, longitudinal sleep study among college students at a private, predominantly white university in a large urban area in the northeastern United States. The current sample consists of 451 college students (Asian = 19%, Black = 15%, Latinx = 19%, multiracial = 23%, white = 24%; female = 76%, male = 22%, non-binary = 2%) who were recruited in the first semester of their first year in college. Groups represented in the multiracial subgroup included white and Latinx (33%), Black and Latinx (20%), white and Asian (13%), Black and white (6%), Asian and Latinx (5%) and Black and Asian (4%). The mean age of the sample during the first fall semester was 18.44 (*SD =* 0.44). A primary aim of the study was to explore differences in a variety of sociodemographic characteristics, such as ethnicity and race, gender, socioeconomic status, first-generation status, and commuter status. Thus, participants were purposefully sampled with these groups in consideration. Of the current sample, 34% were Pell recipients, 34% were first-generation students, and 31% were commuter students.

#### Procedure

Data were collected every fall and spring semester to track changes in sleep-related patterns during college. To ensure a sufficiently large and diverse sample, participants were recruited across three successive cohorts: the first cohort in Fall 2021 (C1, Class of 2025), the second cohort in Fall 2022 (C2, Class of 2026), and the third cohort in Fall 2023 (C3, Class of 2027). The study protocol was approved by the university’s institutional review board, and informed consent was obtained from all participants.

All first-year students were invited to participate and screened for eligibility. Students who were international students or had a medically documented sleep disorder were excluded due to the potential systematic effects on their sleep patterns. At the beginning of the fall and spring semesters, eligible participants were invited to complete an online survey (average response time = 32.40 min; SD = 10.20 min). Participants who completed at least 90% of the survey were subsequently invited to take part in a 14-day daily diary and actigraphy protocol.

During this 14-day period, participants wore wrist actigraphs (Philips Actiwatch Spectrum Plus and ActiGraph CentrePoint Insight Watch) 24 h a day to objectively assess sleep. Over the same 14 days, participants completed a brief daily diary survey each evening before going to bed, reporting on their prior night’s sleep (average response time = 12.04 min; SD = 13.14 min). The survey was distributed at 7:30 PM. Reminder text messages were sent at 11:00 PM if the daily diary had not been completed. Participants who did not complete the diary by 5:00 AM were notified that the completion window for that day had closed. Identical procedures were implemented during all semesters.

The present study used survey data and wrist actigraphy data collected from college students to examine changes in sleep across the first two years of college and to assess potential ethnic and racial disparities in sleep trajectories. Ethnic and racial group membership was assessed using survey data collected only at Time 1 (the first-year fall semester). Daily diary and actigraphy data were collected at four time points spanning two academic years: Time 1 (first-year fall semester), Time 2 (first-year spring semester), Time 3 (second-year fall semester), and Time 4 (second-year spring semester). All survey and daily diary data were collected online via Qualtrics.

#### Measures

Sleep data were obtained using the Philips Actiwatch Spectrum Plus or the ActiGraph CentrePoint Insight Watch across 14 consecutive nights. Wrist actigraphy was used to assess sleep–wake patterns and was set to record 1-minute epochs using Sadeh’s algorithm. Trained research assistants manually identified the bedtime and risetime using an identical visual-inspection procedure. Specifically, using 1-minute epoch activity counts, bedtime was identified as the last three consecutive high-activity epochs (≥ 100 counts/epoch) preceding a sustained decline in activity, and risetime was identified as the first three consecutive high-activity epochs following the low-activity period, consistent with prior validated coding procedures [11, 33, 34]. To establish interrater reliability (IRR), research assistants independently coded the same five practice files, and IRR was evaluated using intraclass correlations. Coding discrepancies were reviewed, and files were re-coded until acceptable reliability was reached (intraclass correlations = 0.89–0.96) for the practice files. After achieving reliability, coders were allowed to code files independently, and every 10th file was double-coded to monitor and prevent rater drift over time. Overall, files were coded with high reliability, with a mean IRR of 0.94. The Philips Actiwatch was discontinued in January 2024, so data collected in the Fall of 2024 were collected on the ActiGraph CentrePoint Insight Watch. As a result, Cohort 3 wore the ActiGraph Centrepoint Insight Watch at Times 3 and 4, whereas all other cohorts and time points used the Philips Actiwatch. Data equivalence was tested across both devices, achieving reliability coefficients of *r* =.997 for bedtime, *r* =.540 for risetime, *r* =.207 for sleep efficiency, and *r* =.954 for total sleep time. Data were downloaded, and sleep periods were coded by trained research assistants, with coding verified against self-reported sleep and wake times from participants’ daily diaries. The coding procedures were identical across the two actigraphy devices, with one additional standardized step required for the ActiGraph CentrePoint Insight Watch. Specifically, after examining differences between the two devices, we identified that the CentrePoint Insight software does not differentiate between sleep offset (i.e., the final epoch scored as sleep, indicating physiological awakening) and risetime (i.e., the time at which the participant physically leaves bed). This distinction is critical for sleep indicators central to the present study, including sleep duration, sleep efficiency, and risetime. We implemented a standardized post-processing coding procedure to differentiate sleep offset and risetime for CentrePoint Insight Watch data, enabling comparability with Philips Actiwatch outputs. Additional details about the procedures used to equate the data can be found in [35].

Bedtime reflects the time at which participants went to bed, separate from the actual time of sleep onset. This variable was manually coded by trained research assistants based on visual inspection of activity counts as the final minutes of activity preceding sustained inactivity at night, operationalized as the last activity count within the final three consecutive minutes of elevated activity (activity count > 100) prior to a sharp and sustained decrease in movement.

Risetime, also referred to as out-of-bed time, represents the time at which participants physically exited bed after awakening. This variable was manually coded by trained research assistants and was indicated by a sharp and sustained increase in activity counts in the morning. It was operationalized as the first activity count within the first three consecutive minutes of elevated activity following the final sleep period.

Sleep duration reflects the total amount of time participants were asleep during a marked rest interval, excluding wake minutes after sleep onset (WASO). Sleep duration was automatically generated by the actigraphy software after coders identified and labeled bedtime and risetime.

Sleep efficiency is an indicator of sleep quality. The sleep efficiency was calculated as: sleep duration/rest duration * 100%, where rest duration includes sleep duration, latency, WASO, and inertia. For the Philips Actiwatch data, sleep efficiency was automatically generated by the Actiware software. For ActiGraph CentrePoint Insight Watch data, sleep efficiency was recalculated after differentiating the sleep offset and risetime to ensure consistency and comparability across devices.

#### Analyses

The first aim of the study was to investigate longitudinal trajectories of sleep outcomes. Latent growth curve modeling (LGCMs [36]) were used to examine trajectories for the full sample, with four timepoints total (e.g., fall and spring semesters for both academic years). Univariate LGCMs were generated using the ‘*lavaan*’ package in RStudio (ver. 2024.04.2 + 764 [37]) and were fit to intercept-only (y = β_0_), linear (y = β_0_ + β_1_x) for all four sleep dimensions. Intercept-only models used three parameters: intercept mean, intercept variance, and residual variance. Factor loadings were to 1 for all timepoints. Linear models used six parameters: intercept mean, slope mean, intercept variance, slope variance, intercept-slope covariance, and residual variance. Slope factor loadings were set at 0, 1, 2, and 3, and assigned to each of the four semesters of college representing linear change. Full maximum likelihood (FIML) estimation was applied to the model to handle potential missing data, aligned with previous work using LGCMs [38, 39].

The second aim of the study was to explore ethnic and racial differences in the trajectories of sleep outcomes. To address this, multigroup LGCMs were estimated with ethnicity and race included as a grouping variable for all four sleep dimensions. Five ethnic and racial categories were included in the analyses (Asian, Black, Latinx, multiracial, white) and separate intercept/slope means were freely estimated. Furthermore, to test ethnic and racial differences in initial (time 1) levels, slope coefficients, and time 4 levels, pairwise Wald chi-square tests were conducted by comparing the latent intercept means and latent slope means across all possible group pairs. Wald test results allowed for specific group differences in both initial sleep levels, change over time, and time 4 levels. A *p*-value of < 0.05 indicated significant differences between two groups.

## Results

Table 1 presents results from all latent growth curve models.

### Aim 1: Sleep Trajectories Among College Students

Profile plots and LGCMs suggest an increase in sleep duration, earlier bedtimes, later risetimes, and declines in efficiency across two years for the full sample. On average, LGCMs indicate that participants reported an initial duration of 7 h and 19 min of sleep, and an average increase of 7.2 min in duration per semester (*β* = 0.12, *SE* = 0.04, *p* <.001). Initial bedtime and risetime time suggested that college students went to bed at 12:58 AM and got out of bed at 8:54 AM on average. The models also indicated that students begin to go to bed earlier and get out of bed later over time, which likely contributed to the increase in duration over time. Slope coefficients indicated that students went to bed an average of 4 min earlier (*β* =−3.95, *SE* = 1.29, *p* <.01) per semester and got out of bed an average of 7 min later (*β* = 7.40, *SE* = 1.46, *p* <.001) per semester. Finally, students had an initial sleep efficiency of 82.59% (*SE* = 0.24, *p* <.001), which declined by 0.73% (*SE* = 0.10, *p* <.001) on average per semester.

### Aim 2: Ethnic and Racial Differences in Sleep Trajectories

**Fig. 1:**
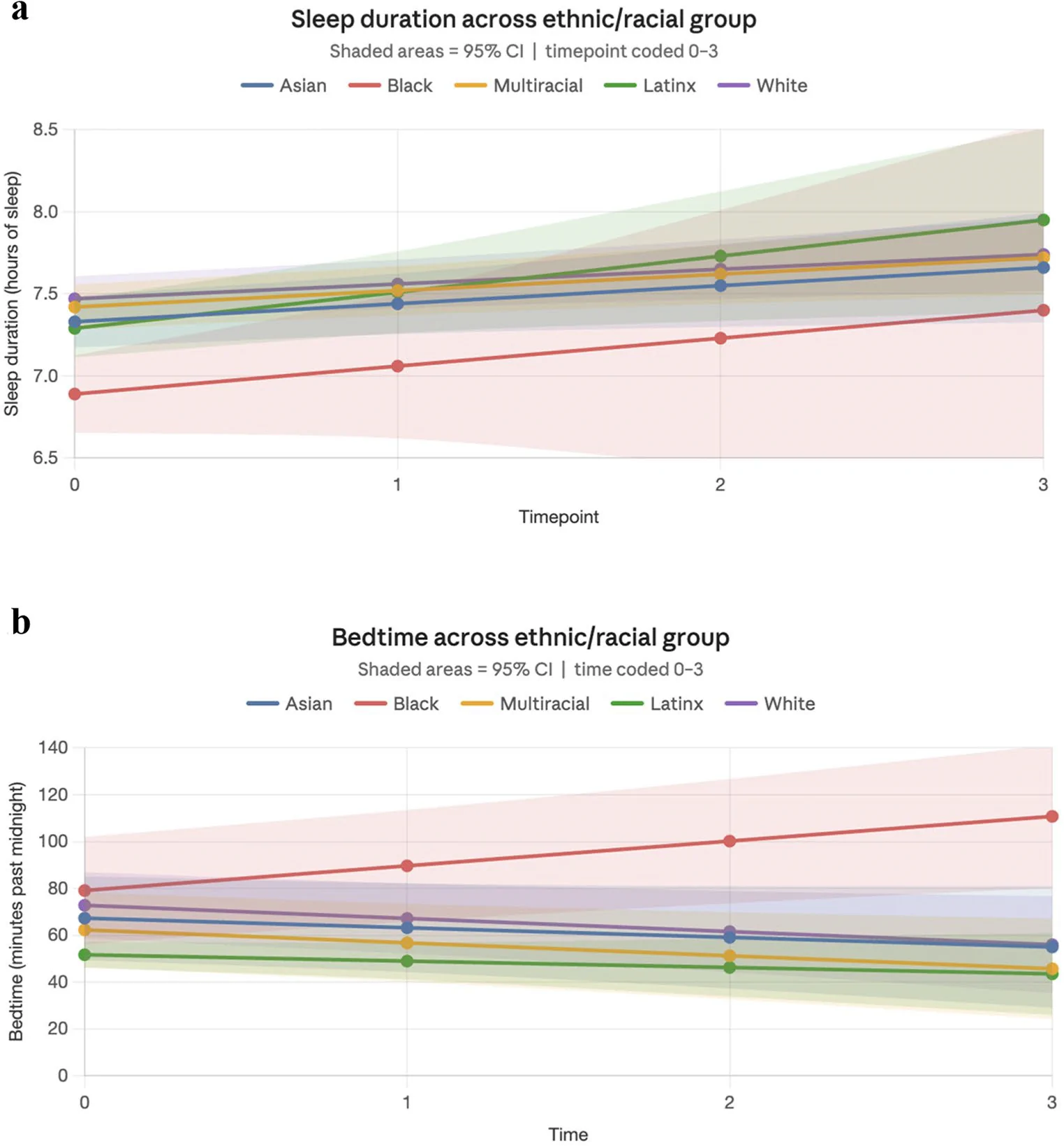

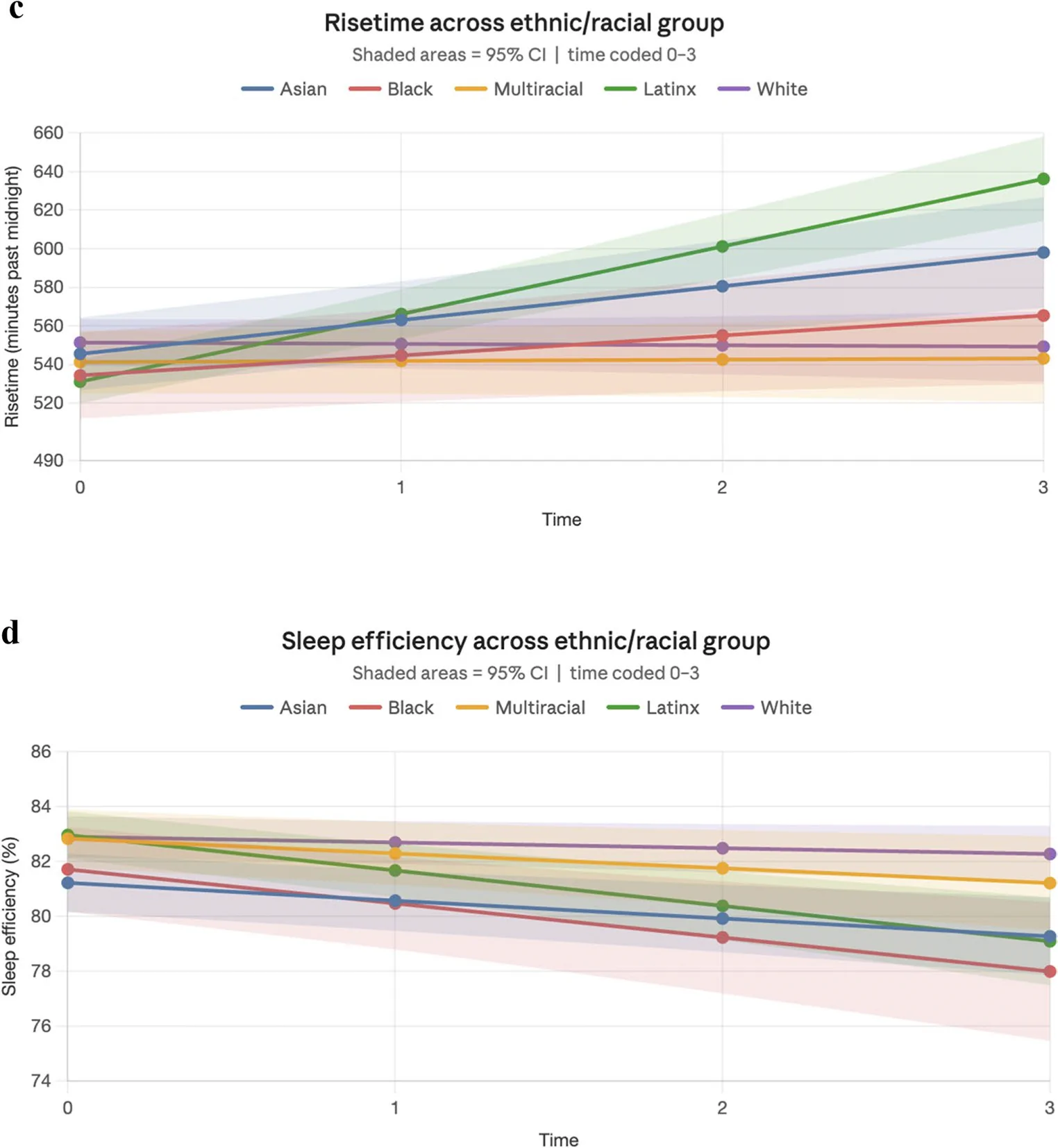
Latent Growth Curve Models by Ethnicity and Race

All results for latent growth curve models across ethnicity and race can be found in Table 2. LGCMs for sleep duration highlighted both consistent disparities as well as some convergence over time. Black students slept significantly less than all other groups during the first fall semester and also had a non-significant increase. All other groups had a significant increase in sleep duration, Latinx students had a significantly faster increase (*β* = 0.22, *SE* = 0.09, *p* <.05) compared to multiracial (*β* = 0.08, *SE* = 0.03, *p* <.01) and white (*β* = 0.09, *SE* = 0.03, *p* <.01), but not Asian (*β* = 0.11, *SE* = 0.05, *p* <.05) or Black (*β* = 0.17, *SE* = 0.09, *p* =.37) students (Table 3). The disparity between Asian and Black students disappeared over time, such that by the spring semester of their sophomore year, Black students had significantly less sleep duration compared to multiracial, white, and Latinx, but not Asian students (Fig. 1a).

Examining bedtime characteristics (bedtime, risetime) revealed unique ethnic and racial disparities emerging over time. Bedtime did not differ by ethnic and racial group during the first fall semester. However, there were significantly earlier bedtimes for multiracial (*β* = −4.24, *SE* = 2.44, *p* <.05) and white (*β* = −7.27, *SE* = 2.56, *p* <.01) students over time. Asian and Latinx students also went to bed earlier, although this change was not statistically significant. Black students went to bed later over time, but this was also not statistically significant. Still, by the spring semester of their sophomore year, Black students had significantly later bedtimes compared to all other ethnic and racial groups (Fig. 1b). There were ethnic and racial differences in risetime for the first semester such that Asians woke up later than Black students, and white students woke up later than Latinx students. There were also within-group increases in risetime (i.e., woke up later) over time, for Latinx (*β* = 14.51, *SE* = 3.19, *p* <.001) and Asian students, although this increase was only marginally significant for Asian students (*β* = 6.32, *SE* = 3.70, *p* <.10). Black and multiracial students had a non-significant increase in risetime, and white students had a non-significant decline (i.e., got out of bed earlier). Latinx students had a significantly faster increase in risetime compared to Asian, white, and multiracial students (but not Black students) such that by the spring semester of sophomore year, Latinx students had significantly later risetimes compared to white and multiracial students, but not Asian and Black students (Fig. 1c). Asian students also had significantly faster increases in risetimes compared to multiracial and Black students such that by the end of sophomore year, Asian students had significantly later risetimes compared to multiracial students.

Modeling sleep efficiency also revealed ethnic and racial disparities emerging over time. For example, although there were no significant ethnic and racial differences in sleep efficiency during the first fall semester, disparities emerged such that only ethnic and racially minoritized students had a significant decline (poorer sleep efficiency over time), with white students having a non-significant decline (Table 1). Black students had faster declines in sleep efficiency (*β* = −1.24, *SE* = 0.34, *p* <.001) compared to Asian (*β* = − 0.65, *SE* = 0.16, *p* <.001) and multiracial (*β* = − 0.54, *SE* = 0.23, *p* <.05) students, but not white (*β* = − 0.21, *SE* = 0.12, *p* =.10) or Latinx students (*β* = −1.29, *SE* = 0.23, *p* <.001). Asian students’ trajectory fell in between, having significantly faster declines in sleep efficiency compared to white students and multiracial students, but a significantly slower decline compared to Latinx and Black students. Finally, Latinx students had faster declines than white and multiracial students, slower declines than Asian students, and were statistically indistinguishable from Black students. By the second semester of sophomore year, white and multiracial students had significantly greater sleep efficiency compared to Latinx and Asian students. The difference in sleep efficiency between white and multiracial students was not significant.

## Discussion

The current study examined longitudinal sleep trajectories in a diverse sample of first-year college students across the first two years of college, with a specific focus on sleep duration, bedtime characteristics (bedtime, risetime), and sleep efficiency. In line with our hypotheses and existing data, sleep dimensions significantly changed over time, and plotting trajectories revealed ethnic and racial disparities emerging and persisting across multiple dimensions of sleep. Altogether, these results underscore sleep as a dynamic health behavior with important implications for ethnic and racial health disparities among college students.

### Aim 1: Sleep Trajectories Among College Students

Investigating multiple sleep dimensions (duration, bedtime, risetime, and efficiency) using actigraph data was a major strength of the study. Looking at these dimensions together presents implications for sleep behaviors over time. Overall, college students gained more average sleep over time, which corresponded to going to bed earlier and getting out of bed later. This finding contributes to the literature by supporting earlier works that find sleep duration to increase during emerging adulthood [40]. Changes in bedtime and risetime suggest that over time college students begin to adapt to their environments and make necessary adjustments to their bedtime schedules [18]. Changes in living situations (e.g., housing, roommates) in addition to different class schedules can allow for earlier bedtimes and later risetimes that increase sleep duration. Furthermore, greater familiarity with college-related stressors between the first and second year of college may also explain improvements in sleep duration over time. Qualitative research suggests that college students initially face challenges with self-directed learning skills but develop these skills during their first year [41, 42]. Improved learning skills may result in improved time management that facilitates earlier bedtimes and later risetimes corresponding with longer sleep duration [42].

However, in spite of this increase in sleep duration, college students in the current study also suffered from an average decline in sleep efficiency during the same time period, a finding consistent with prior research [18–20]. Replicating similar findings when looking at just the transition to college [17], students may be getting *sufficient* sleep, but still struggle to maintain *quality* sleep, potentially compromising the restorative benefits. This study extends the prior research by demonstrating that this discrepancy intensifies over time. Even as college students gain more sleep over time, their quality of sleep continues to worsen. Altogether, these findings emphasize the need for interventions on college campuses that move beyond promoting longer sleep duration but also consistently target sleep quality [43]. Prioritizing factors that are associated with poor sleep quality, such as stress, noise, and irregular schedules, instead of targeting sleep duration alone may improve college student sleep health and daily functioning [44].

### Aim 2: Ethnic and Racial Differences in Sleep Trajectories

The current study also provided a strong basis that these sleep trajectories differed across ethnic and racial groups, demonstrating that disparities persisted and even widened over time. Overall, in support of our hypotheses and in line with prior research [23], white students consistently experienced more favorable sleep trajectories over time compared to all other groups. In contrast, Black students experienced consistent disparities in sleep, and Asian, Latinx, and multiracial students consistently fell between, replicating earlier findings documenting similar patterns among ethnic and racial minority youth [24, 26].

Asian students demonstrated intermediate sleep trajectories that, in some cases, converged with those of other marginalized groups over time. For example, the initial disparity in sleep duration between Asian and Black students disappeared over time, such that by the spring of sophomore year these groups’ levels of sleep duration were indistinguishable. Additionally, disparities emerged in sleep efficiency such that Asian students experienced a significant decline such that they were significantly lower compared to white and multiracial students, but indistinguishable from Black and Latinx students by the end of their sophomore year. Their decline in sleep was faster than that of white students, though slower than that of Black students, and indistinguishable from the decline among Latinx and multiracial students. Overall, the findings suggest that Asian students may not exhibit sleep disadvantages during the transition to college but may become increasingly susceptible to declines in sleep efficiency as college progresses, although it remains unclear what may drive this decline for Asian students compared to other ethnic and racial minority youth [17]. Future research should explore factors that may explain the decline in sleep efficiency among Asian students.

Observed disparities for Black students were largely consistent with existing research [45], exhibiting the most consistent and persistent sleep disadvantage across the college years. Black students had significantly less sleep duration compared to all other groups during the first semester of college and, unlike other groups, experienced no increase in sleep duration. However, it is worth noting that the standard error for Black students (*SE* = 0.19) was higher than other groups, indicating large variability for Black students. This large variability may be explaining why the increase was not significant, despite the slope coefficient (*β =* 0.17) being higher than other groups. Moreover, although the disparity between Black and Asian students narrowed, Black students continued to experience significantly shorter sleep duration than multiracial, white, and Latinx students by the spring of their sophomore year.

Furthermore, despite going to bed at similar times as other groups during the first semester of college, Black students were the only group to go to bed later (although this was non-significant) compared to all other groups who tended to go to bed earlier into the second year of college. This resulted in Black students having later bedtimes compared to all other groups by the end of sophomore year. It is particularly striking that the Black students’ only significant change in two years was a decline in sleep efficiency, which was also a significantly faster decline compared to white, Asian, and multiracial (but not Latinx) students. Of note, one study among younger children found that Black children had later bedtimes compared to white children but had similar risetimes [46]. This consistency in going to bed later and sharp decline in sleep efficiency may reflect unique stressors experienced by Black students. Prior research has found that Black college students may experience higher rates of discrimination, even compared to other ethnic and racial groups [45, 47, 48]. Once again, further research should explore the specific factors that may explain later bedtimes among Black college students.

The sleep patterns observed among Latinx students suggest disparities related to duration, quality, and risetime. Latinx students had the longest sleep duration by the end of sophomore year, significantly more than Black students, which was driven by the fastest increase in sleep duration (*β =* 0.17) compared to multiracial and white students. Although Latinx students remained consistent in their going to bed across two years, they were the only group in the sample to experience a change in risetime such that they got out of bed later every semester. This resulted in Latinx students waking up later compared to Asian, multiracial, and white students by the end of sophomore year. These findings build on existing research that observes earlier bedtimes and risetimes among Latinx students compared to other ethnic and racial groups [17]. It is possible that consistent bedtime routines that are emphasized in parental socialization among Latinx families may explain these trajectories [49], although future research should continue to explore other academic or cultural factors. Finally, despite increases in sleep duration, Latinx students experienced one of the fastest declines in sleep efficiency. This decline was significantly faster than that of white and multiracial students and indistinguishable from that of Asian and Black students. Similar to Asian students, research should explore factors that may uniquely explain the decline in sleep efficiency among Latinx students.

The systematic inclusion of multiracial students was a key contribution of the study. These students experienced increasingly longer sleep duration, which was accompanied with earlier bedtimes and consistent risetimes. Although multiracial students experienced declines in sleep efficiency, these declines were slower than those among Black and Latinx students such that by the end of sophomore year, multiracial students had significantly better sleep efficiency compared to Black and Latinx students and were indistinguishable from white students. Once again, these findings replicate earlier findings that find multiracial students having relatively good sleep quality [17]. However, findings for multiracial students’ sleep outcomes are mixed, with newer research finding that experiences of discrimination may still result in poorer sleep health for this group [50, 51]. These mixed findings support the growing effort to include multiracial status when conducting sleep disparity research. As multiracial students make up a heterogeneous group of racial and ethnic identities (e.g., Latinx and Black, Latinx and white, Asian and white), future research should examine within-group variation based on ethnic/racial identity, phenotypical characteristics, and experiences of discrimination [51].

As predicted by the existing literature, white students overall reported more favorable sleep outcomes over time compared to ethnic and racial minorities, replicating earlier findings [17, 23]. Similar to multiracial students, white students experienced an increase in sleep duration, coupled with earlier bedtimes and consistent risetimes across two years. Notably, despite no significant differences in sleep efficiency compared to other groups during the transition to college, white students were the only group who did not experience a decline in sleep efficiency. As a result, by the end of sophomore year white students had greater sleep efficiency compared to Asian, Black, and Latinx students (but were indistinguishable from multiracial students).

### Bedtime Characteristics and Sleep Efficiency

The current study also offers unique insight into what may be driving observed disparities by exploring multiple dimensions of sleep, including duration, bedtime characteristics (bedtime, risetime), and sleep efficiency using actigraph data (see Table 4 for a summary of changes in sleep across ethnic and racial groups). For example, exploring these variables together suggested that the increase in sleep duration was driven by different bedtime behaviors across ethnic and racial groups. Among multiracial and white students, who showed relatively favorable sleep patterns, increases in sleep duration seem to be driven by *going to bed earlier* over time. In contrast, Latinx and Asian students also experienced an increase in sleep duration but seem to be driven from *waking up later* over time. These findings underscore a novel finding of the study and emphasize the benefit of exploring multiple dimensions of sleep to uncover unique patterns that delineate ethnic and racial disparities.

It is worth noting that the multiracial and white students, whose increased sleep duration stemmed from earlier bedtime, had the best sleep efficiency, whereas Latinx and Asian students, whose increased sleep duration stemmed from later risetimes, had the poorest sleep efficiency, significantly lower than multiracial and white students. Furthermore, Black students, who had the latest bedtime (and second latest risetime) had the poorest sleep efficiency, significantly worse than multiracial and white students. Together, these patterns suggest that although going to bed earlier or waking up later can independently drive increases in sleep duration, going to bed earlier (opposed to waking up later) seems to be a better practice for maintaining sleep efficiency over time. This novel finding contributes to ongoing research exploring the role of sleep timing and sleep quality among adults [52].

### Limitations and Future Directions

There are some limitations worth considering when interpreting this data. First, the current sample consisted of undergraduate students enrolled in one university, limiting the generalizability of these findings. This is particularly worth noting since this university is a private university in a metropolitan city. As such, the current sample may have unique student experiences that may influence sleep patterns, such as increased urbanicity (i.e., noise exposure, light exposure) and commuting. These factors may not be as prevalent in other academic settings. Thus, more research is needed to investigate ethnic and racial disparities in sleep over time in more generalized settings, such as in public universities or in non-industrialized regions. Expanding these findings across diverse educational contexts can help elucidate the generalizability of ethnic and racial sleep disparities across different populations.

In addition, the current study is limited to descriptive findings. Thus, no causal inferences can be made about the underlying mechanisms between ethnic and racial group membership and sleep outcomes. Still, these findings provide a foundation for identifying when and where ethnic and racial disparities emerge across different sleep dimensions and timepoints. Future aims of the parent study will examine potential mediating variables, such as discrimination stress, that may explain the link between ethnic and racial group membership and sleep health. Examining these pathways using longitudinal analyses will be particularly valuable in discovering causal pathways, which can further inform intervention strategies to address these disparities.

## Conclusion

Entering college is a major transition with a host of environmental stressors. Understanding the sleep health of new college students during their first years of college can be beneficial for identifying when potential disparities in sleep may emerge, as well as how these disparities look over time. The current longitudinal study explored ethnic and racial disparities in a sample of U.S. college students during their first two years of college and used actigraph data to capture multiple dimensions of sleep. In support of our hypothesis, white students consistently reported the most favorable outcomes across the first two years relative to ethnic and racial minority students, whereas Black students consistently reported disparities in sleep. Asian, Latinx, and multiracial students showed sleep patterns that fell between these two groups. In addition, although there were shared trends in sleep improvements over time, such as increased sleep duration, there were notable differences in trajectories of bedtime characteristics and sleep efficiency across ethnic and racial group membership. Overall, this research highlights college students’ diverse sleep patterns when adjusting to college environments while also underscoring the need to consider ethnic and racial disparities over time.

## Supplementary Material

S Table 1

**Supplementary Information** The online version contains supplementary material available at https://doi.org/10.1007/s40615-026-03091-y.

## Figures and Tables

**Table 1:** Latent growth curve models for the full sample

	Sleep Duration		Bedtime		Risetime		Sleep Efficiency	
			
Parameters	Estimate	SE	Estimate	SE	Estimate	SE	Estimate	SE

Intercept mean	7.28***	0.04	58.42***	3.85	534.66***	3.64	82.59***	0.24
Intercept residual variance	0.84*	0.38	3806.24	426.22	3297.03*	374.24	14.32***	1.75
Slope mean	0.12**	0.04	−3.95**	1.29	7.40***	1.46	−0.73***	0.10
Slope residual variance	0.12	0.35	202.14	57.26	357.34	65.58	2.18***	0.30
Intercept and slope covariance	− 0.35	0.37	−191.29	120.73	−289.66*	124.97	−1.28*	0.55

Note

* *p* <.05,

** *p* <.01,

*** *p* <.001

**Table 2:** Latent growth curve models unstandardized estimates for sleep outcomes across ethnic and racial groups

Sleep Duration	Asian		Black		Latinx		Multiracial		White	
Parameters	Estimate	SE	Estimate	*SE*	Estimate	*SE*	Estimate	*SE*	Estimate	*SE*
Intercept mean	7.22***	0.08	6.89***	0.012	7.29***	0.09	7.41***	0.08	7.47***	0.07
Intercept residual variance	0.52**	0.15	0.75	0.74	0.84	1.37	0.25*	0.10	0.43***	0.09
Slope mean	0.11*	0.05	0.17	0.19	0.22*	0.09	0.08**	0.03	0.09**	0.03
Slope residual variance	0.08	0.05	4.19*	1.83	0.36	1.18	0.00	0.02	0.01	0.02
Intercept and slope covariance	−0.11	0.08	−0.42	0.78	− 0.42	1.34	0.04	0.03	0.02	0.04
Bedtime	Asian		Black		Latinx		Multiracial		White	
Parameters	Estimate	SE	Estimate	*SE*	Estimate	*SE*	Estimate	*SE*	Estimate	*SE*
Intercept mean	70.14***	9.12	65.76***	11.68	45.41***	2.83	53.99***	8.14	58.16***	7.21
Intercept residual variance	4901.76***	1123.41	2490.63	1320.22	3244.04***	846.21	4723.45***	1009.05	2829.45***	682.68
Slope mean	−4.03	3.20	1.07	3.43	−1.16	2.83	−4.24*	2.44	−7.27**	2.56
Slope residual variance	416.22**	143.96	108.56	542.74	40.10	221.64	293.68**	103.18	161.83	100.46
Intercept slope covariance	−247.63	306.61	449.74	446.09	−235.76	287.22	−533.32*	258.67	−129.75	204.15
Risetime	Asian		Black		Latinx		Multiracial		White	
Parameters	Estimate	SE	Estimate	*SE*	Estimate	*SE*	Estimate	*SE*	Estimate	*SE*
Intercept mean	542.25***	9.59	518.48***	11.38	518.45***	5.76	537.02***	8.26	545.81***	6.15
Intercept residual variance	5040.18***	1122.32	2657.44*	1083.96	945.44	511.88	4479.97***	1018.51	2152.08***	495.26
Slope mean	6.32+	3.70	1.35	4.69	14.51***	3.19	3.88	2.72	1.32	2.33
Slope residual variance	454.37*	184.45	338.21	264.60	381.66**	140.28	229.07	121.17	165.10*	80.89
Intercept slope covariance	−299.48	349.55	41.11	423.04	120.95	195.10	−392.82	287.03	−285.92	165.28
Sleep Efficiency	Asian		Black		Latinx		Multiracial		White	
Parameters	Estimate	SE	Estimate	*SE*	Estimate	*SE*	Estimate	*SE*	Estimate	*SE*
Intercept mean	82.06***	0.54	81.69***	0.79	82.97***	0.44	82.86***	0.54	82.91***	0.38
Intercept residual variance	13.50***	3.82	21.67**	6.25	14.34***	3.83	15.32***	4.35	6.38**	1.99
Slope mean	− 0.65***	0.16	−1.24***	0.34	−1.29***	0.23	− 0.54*	0.23	− 0.21	0.12
Slope residual variance	0.27	0.48	2.99*	1.16	3.83***	0.71	2.64**	0.79	0.19	0.37
Intercept slope covariance	0.89	1.01	−2.32	2.11	−2.51*	1.09	−1.58	1.39	0.53	0.68

Note.

+ *p* <.10,

* *p* <.05,

** *p* <.01,

*** *p* <.001

**Table 3:** Pairwise comparisons across sleep dimensions

Contrast	χ^2^	*p*-value

T1 Duration		
Black vs. white	9.44**	< 0.01
Black vs. Latinx	4.49*	< 0.05
Black vs. Asian	5.94*	< 0.05
Black vs. multiracial	11.91***	< 0.001
Asian vs. white	0.85	0.36
Asian vs. Latinx	0.09	0.76
Asian vs. multiracial	1.90	0.17
Latinx vs. white	1.34	0.25
Latinx vs. multiracial	2.52	0.11
white vs. multiracial	0.15	0.70
Slope Duration		
Black vs. white	0.55	0.46
Black vs. Latinx	0.53	0.47
Black vs. Asian	0.40	0.53
Black vs. multiracial	0.66	0.42
Asian vs. white	0.00	0.99
Asian vs. Latinx	2.53	0.11
Asian vs. multiracial	0.01	0.92
Latinx vs. white	4.03*	< 0.05
Latinx vs. multiracial	4.25*	< 0.05
white vs. multiracial	0.01	0.91
T4 Duration		
Black vs. white	5.71*	< 0.05
Black vs. Latinx	9.37**	< 0.01
Black vs. Asian	2.53	0.11
Black vs. multiracial	6.64**	< 0.01
Asian vs. white	0.49	0.48
Asian vs. Latinx	2.06	0.15
Asian vs. multiracial	0.80	0.37
Latinx vs. white	0.67	0.41
Latinx vs. multiracial	0.38	0.54
white vs. multiracial	0.05	0.83
T1 Bedtime		
Black vs. white	1.51	0.22
Black vs. Latinx	3.99	0.05
Black vs. Asian	1.02	0.31
Black vs. multiracial	2.05	0.15
Asian vs. white	0.02	0.88
Asian vs. Latinx	1.22	0.27
Asian vs. multiracial	0.18	0.67
Latinx vs. white	1.24	0.27
Latinx vs. multiracial	0.56	0.46
white vs. multiracial	0.11	0.74
Slope Bedtime		
Black vs. white	5.84*	< 0.05
Black vs. Latinx	2.02	0.16
Black vs. Asian	3.12	0.08
Black vs. multiracial	6.27*	< 0.05
Asian vs. white	0.16	0.69
Asian vs. Latinx	0.24	0.62
Asian vs. multiracial	0.25	0.62
Latinx vs. white	1.04	0.31
Latinx vs. multiracial	1.26	0.26
white vs. multiracial	0.01	0.91
		
T4 Bedtime		
Black vs. white	9.89**	< 0.01
Black vs. Latinx	9.92**	< 0.01
Black vs. Asian	5.22*	< 0.05
Black vs. multiracial	12.08***	< 0.001
Asian vs. white	0.22	0.64
Asian vs. Latinx	0.26	0.61
Asian vs. multiracial	0.65	0.42
Latinx vs. white	0.00	0.96
Latinx vs. multiracial	0.14	0.70
white vs. multiracial	0.19	0.66
**T1 Risetime**		
Black vs. white	0.10	0.75
Black vs. Latinx	0.01	0.94
Black vs. Asian	4.51*	< 0.05
Black vs. multiracial	1.61	0.20
Asian vs. white	0.08	0.78
Asian vs. Latinx	1.79	0.18
Asian vs. multiracial	0.77	0.38
Latinx vs. white	5.50*	< 0.05
Latinx vs. multiracial	1.64	0.20
white vs. multiracial	0.02	0.90
**Slope Risetime**		
Black vs. white	0.31	0.58
Black vs. Latinx	0.39	0.53
Black vs. Asian	11.55***	< 0.001
Black vs. multiracial	16.30***	< 0.001
Asian vs. white	1.16	0.28
Asian vs. Latinx	5.56*	< 0.05
Asian vs. multiracial	10.81**	< 0.01
Latinx vs. white	16.50***	< 0.001
Latinx vs. multiracial	20.15***	< 0.001
white vs. multiracial	2.40	0.12
T4 Risetime		
Black vs. white	0.05	0.82
Black vs. Latinx	0.49	0.49
Black vs. Asian	2.69	0.10
Black vs. multiracial	7.78**	< 0.01
Asian vs. white	0.57	0.45
Asian vs. Latinx	1.14	0.29
Asian vs. multiracial	5.20*	< 0.05
Latinx vs. white	6.12*	< 0.05
Latinx vs. multiracial	11.25***	< 0.001
white vs. multiracial	2.62	0.11
T1 Efficiency		
Black vs. white	1.16	0.28
Black vs. Latinx	0.02	0.89
Black vs. Asian	0.02	0.89
Black vs. multiracial	1.34	0.25
Asian vs. white	1.05	0.31
Asian vs. Latinx	1.02	0.31
Asian vs. multiracial	0.09	0.77
Latinx vs. white	0.00	0.99
Latinx vs. multiracial	1.23	0.27
white vs. multiracial	1.22	0.27
Slope Efficiency		
Black vs. white	0.01	0.94
Black vs. Latinx	2.75	0.10
Black vs. Asian	5.71*	< 0.05
Black vs. multiracial	6.27*	< 0.05
Asian vs. white	5.55*	< 0.05
Asian vs. Latinx	7.84**	< 0.01
Asian vs. multiracial	7.31**	< 0.01
Latinx vs. white	23.14***	< 0.001
Latinx vs. multiracial	15.98***	< 0.001
white vs. multiracial	0.52	0.47
T4 Efficiency		
Black vs. white	0.89	0.34
Black vs. Latinx	2.33	0.13
Black vs. Asian	6.37*	< 0.05
Black vs. multiracial	9.17**	< 0.01
Asian vs. white	7.58**	< 0.01
Asian vs. Latinx	2.77	0.10
Asian vs. multiracial	6.04*	< 0.05
Latinx vs. white	20.85***	< 0.001
Latinx vs. multiracial	17.43***	< 0.001
white vs. multiracial	1.85	0.17

Note.

* *p* <.05,

** *p* <.01,

*** *p* <.001

**Table 4:** Summary of sleep changes across ethnic and racial groups over two years

	Asian	Black	Multiracial	Latinx	White

Duration	**Improves**	No change	**Improves**	**Improves**	**Improves**
Bedtime	No change	No change	**Improves**	No change	**Improves**
Risetime	No change	**Improves**	No change	**Improves**	No change
Efficiency	**Worsens**	**Worsens**	**Worsens**	**Worsens**	No change

## Data Availability

Data is available upon reasonable request.

## Code Availability

RStudio code is available upon reasonable request.
