# Supplementary material for: Racial Differences in Sleep Outcomes in the First Two Years of College
Source: J Racial Ethn Health Disparities. Author manuscript; Available in PMC 2026 Aug 27. (PMC13521211; doi:10.1007/s40615-026-03091-y)
Supplement: Supplementary file 1 [file NIHMS2198205-supplement-S_Table_1.docx]

**Supplementary Materials**

**Table 1.** Reliability coefficients across watch devices

| Sleep metric | Reliability (r) |
| --- | --- |
| Sleep Duration | .98 |
| Bedtime | .54 |
| Risetime | .21 |
| Sleep Efficiency | .95 |

*Note*. Data were downloaded, and sleep periods were coded by trained research assistants, with coding verified against self-reported sleep and wake times from participants’ daily diaries. The coding procedures were identical across the two actigraphy devices, with one additional standardized step required for the ActiGraph CentrePoint Insight Watch. Specifically, after examining differences between the two devices, we identified that the CentrePoint Insight software does not differentiate between sleep offset (i.e., the final epoch scored as sleep, indicating physiological awakening) and risetime (i.e., the time at which the participant physically leaves bed). This distinction is critical for sleep indicators central to the present study, including sleep duration, sleep efficiency, and risetime.
